# Thermal Plasma Synthesis of Crystalline Gallium Nitride Nanopowder from Gallium Nitrate Hydrate and Melamine

**DOI:** 10.3390/nano6030038

**Published:** 2016-02-24

**Authors:** Tae-Hee Kim, Sooseok Choi, Dong-Wha Park

**Affiliations:** 1 Department of Chemistry and Chemical Engineering and Regional Innovation Center for Environmental Technology of Thermal Plasma (RIC-ETTP), Inha University, 100 Inha-ro, Nam-gu, Incheon 22212, Korea; taehee928@naver.com; 2 Department of Nuclear and Energy Engineering, Jeju National University, 102 Jejudaehak-ro, Jeju 63243, Korea

**Keywords:** thermal plasma, annealing, gallium nitride, gallium nitrate hydrate, melamine, nanopowder

## Abstract

Gallium nitride (GaN) nanopowder used as a blue fluorescent material was synthesized by using a direct current (DC) non-transferred arc plasma. Gallium nitrate hydrate (Ga(NO_3_)_3_∙*x*H_2_O) was used as a raw material and NH_3_ gas was used as a nitridation source. Additionally, melamine (C_3_H_6_N_6_) powder was injected into the plasma flame to prevent the oxidation of gallium to gallium oxide (Ga_2_O_3_). Argon thermal plasma was applied to synthesize GaN nanopowder. The synthesized GaN nanopowder by thermal plasma has low crystallinity and purity. It was improved to relatively high crystallinity and purity by annealing. The crystallinity is enhanced by the thermal treatment and the purity was increased by the elimination of residual C_3_H_6_N_6_. The combined process of thermal plasma and annealing was appropriate for synthesizing crystalline GaN nanopowder. The annealing process after the plasma synthesis of GaN nanopowder eliminated residual contamination and enhanced the crystallinity of GaN nanopowder. As a result, crystalline GaN nanopowder which has an average particle size of 30 nm was synthesized by the combination of thermal plasma treatment and annealing.

## 1. Introduction

Gallium nitride (GaN) has been used as a binary III–V direct band-gap semiconductor material in light-emitting diodes since the 1990s. GaN is a blue fluorescence material used in LEDs. Lighting devices create various colors by combining red (R), green (G), and blue (B) [1]. In order to prepare a white light source, blue fluorescence is mixed with other light sources such as yellow, red, or green light. Such white LEDs are taking the place of traditional incandescent and fluorescent lights. GaN has a large band gap energy of 3.4 eV at room temperature and a high thermal conductivity of 130 W/m∙K [2,3,4]. It is possible to use these materials in optoelectronic devices which have wide band gap with energies from the visible to the deep ultraviolet region. Gallium nitride is a very hard material that has a strong atomic bonding as a wurtzite crystal structure. It can be used for applications in optoelectronic, high-power, high-frequency, and high temperature devices. For example, GaN can be applied as the substrate which makes violet laser diodes at 405 nm without the requirement of nonlinear optical frequency-doubling. It is usually applied by deposition on silicon carbide (SiC) and sapphire (Al_2_O_3_) plates. During the deposition of GaN onto plates, the mismatching of GaN lattice structure could occur, and doping to n-type and p-type materials with silicon or oxygen elements has appeared [5,6]. This mismatch disturbs the crystal growth and leads to defects of crystal GaN due to increased tensile stress. In order to produce an excellent GaN device, crystalline GaN should be grown uniformly on the plate.

In the present industry, gallium nitride is often grown on foreign substrates on thin films by MOVPE (metal organic vapor phase epitaxy) and MOCVD (metal organic chemical vapor deposition). However, the thermal expansion coefficient of GaN is considerably higher than that of silicon or sapphire. These different properties invite a crack of epitaxial films or wafer bowing in the GaN growth process on the substrate. Therefore, it is difficult to produce bulk single crystals.

GaN powder can be produced by a novel hot mechanical alloying process. It requires a lengthy process to yield the powder by this method and the purity of the product is not sufficient [7]. In the methods for synthesis of GaN powder, GaN can be synthesized from molten gallium metal with a stream of reactive NH_3_ gas in a furnace. Gallium metal is maintained at a molten state at 30 °C. However, it is difficult to vaporize at a low temperature due to its high vaporization temperature of 2400 °C. Although this method is simple and economic, it is not suitable to produce quality GaN powder [2,8]. The ammonothermal reduction nitridation method can produce GaN powder by reacting gallium oxide with NH_3_ in the temperature range between 600 and 1100 °C. However, incomplete nitridation of the oxide easily occurs. In other words, the purity of synthesized GaN is fairly low. Gallium phosphide (GaP) and Gallium arsenide (GaAs) are possible alternative materials for Gallium oxide in the temperature range of 1000–1100 °C [2,9]. Carbothermal reduction nitridation is applied to synthesize GaN nanorods and nanowires. Carbon is employed as a catalyst for crystal growth [3]. Therefore, an additional decarbonization process is necessary to eliminate the residual carbon. Liquid precursors can be used by an aerosol-assisted vapor phase synthesis method. It is completed at a relatively low temperature. However, this method has a multitude of steps such as preparation of gallium solid compounds, oxidation of gallium precursor, and nitridation chemistry. Therefore, it takes an extended synthesis time to produce GaN powder [10]. Arc plasma has been regarded as an effective method to obtain nanometer-sized GaN. This method requires a very short time compared with other synthesis methods [11,12]. However, gallium lump is an expensive precursor and its evaporation requires an extended time in the arc plasma region. Generally, conventional synthesis methods have several limitations for the preparation of nano-sized particles and complete nitridation.

GaN has commonly been grown in the industrial field by the epitaxial method on the substrate. However, it is expected that the uniform deposition of GaN is achievable using the nanoparticle printing method rather than the conventional chemical vapor deposition (CVD) method [6]. Therefore, using the new method would mean that the fine particles with a high crystallinity could be printed on a substrate. It was expected that this method would prevent the mismatch of lattice and irregular growth. In this work, thermal plasma which is able to produce GaN fine particles was applied as substitute technology of the conventional CVD method. Gallium nitrate hydrate (Ga(NO_3_)_3_∙*x*H_2_O) was used as the raw material instead of Ga or Ga_2_O_3_ which have been used in previous studies to synthesize GaN [2,3,4,10,13,14,15,16,17,18,19,20]. However, the raw material itself has abundant oxygen elements. Therefore, melamine (C_3_H_6_N_6_) was additionally injected into the thermal plasma jet to prevent the oxidation of decomposed Ga into Ga_2_O_3_ [21,22]. In order to improve the crystallinity of synthesized GaN, products from the thermal plasma were subjected to annealing using a vacuum furnace. The crystallinity and purity of synthesized GaN nanopowder were investigated before and after annealing.

## 2. Experimental Setup

**Figure 1:**
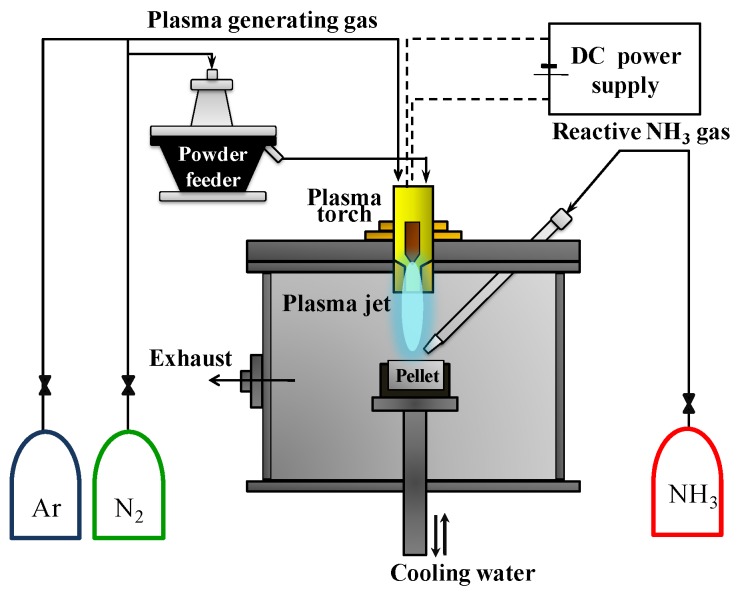
Experimental apparatus for the synthesis of GaN (gallium nitride) nanopowder by DC (direct current) non-transferred thermal plasma.

GaN nanopowder was synthesized from Ga(NO_3_)_3_∙*x*H_2_O (99.9% purity, Alfa Aeser Inc., Boston, MA, USA) and C_3_H_6_N_6_ (99% purity, Ald rich Inc., St. Louis, MO, USA) using the non-transferred DC arc plasma. The schematic diagram of the thermal plasma system is indicated in Figure 1. The system consists of a DC power supply (YC-500TSPT5, Technoserve, Toyohashi, Japan), a plasma torch (SPG-30N2S, Technoserve, Japan), a powder feeder (ME-14C, SHINKO Electric Co Nagoya, Japan) for the injection of the precursor, a chamber, and a crucible for the precursor. The thermal plasma jet was generated in the plasma torch by Ar or Ar–N_2_, which formed gases under atmospheric pressure. The thermal plasma jet was ejected from a 6 mm diameter nozzle by the thermal expansion of plasma, forming gas by an electric arc channel which was connected between a conical cathode and the anode nozzle. Ga(NO_3_)_3_∙*x*H_2_O powder was used as the raw material for the synthesis of GaN nanopowder. The injected precursor powder was hundreds of micrometers in size, and its morphology is not specific. The precursor was a pellet which had a diameter of 45 mm and a thickness of 10 mm created by a hydraulic press. The precursor pellet was placed on a tungsten crucible which was fixed by a water-cooling holder. As a result, the precursor was rapidly melted and evaporated by the confronting thermal plasma jet, as shown in Figure 1. The vaporization temperature of Ga(NO_3_)_3_∙*x*H_2_O is lower than 100 °C. Therefore, it is a more economical precursor than Ga or Ga_2_O_3_ which were used as precursors to synthesize GaN in previous work [23]_._ However, it contains excessive nitrogen, oxygen, and hydrogen elements. The complete dissociation of the oxygen from Ga(NO_3_)_3_∙*x*H_2_O is difficult due to the high latent heat of H_2_O. For this reason, it is normal that evaporated Ga(NO_3_)_3_∙*x*H_2_O is oxidized to Ga_2_O_3_ by its inherent oxygen elements. Therefore, melamine (C_3_H_6_N_6_) powder was used to prevent the production of gallium oxide. The injected C_3_H_6_N_6_ was converted to carbon, nitrogen, hydrogen, and other molecules by decomposition at high temperatures in the thermal plasma jet. These byproduct molecules consume the oxygen elements of the Ga(NO_3_)_3_∙*x*H_2_O precursor. Injected melamine powder consists of nonspecific-shaped particles under 500 nm. In addition, ammonia (NH_3_) gas was diagonally injected into the pellet of Ga(NO_3_)_3_∙*x*H_2_O from the upper side of the chamber to nitride the Ga element.

The detailed operating conditions are indicated in Table 1. In the first case, the condition of Plasma 1, the Ga(NO_3_)_3_∙*x*H_2_O pellet was reacted with only NH_3_ gas and without C_3_H_6_N_6_. The thermal plasma jet was generated by mixed argon and nitrogen gases. The high thermal conductive nitrogen was injected as a plasma forming gas to help the nitridation or evaporation as the input power was increased. The plasma input power was 12.6 kW at the fixed current of 300 A and the average voltage of 42 V. In the other cases of Plasma 2, 3, 4, and 5, the Ga(NO_3_)_3_∙*x*H_2_O pellet was evaporated by pure Ar thermal plasma together with C_3_H_6_N_6_. In these cases, the input power for the generation of the thermal plasma jet was 8.4 kW with the current fixed at 300 A and an average voltage of 28 V. Argon is a monoatomic molecule and nitrogen is a diatomic molecule. In order to generate thermal plasma from nitrogen gas, more energy is required compared to argon gas. Accordingly, electric resistance for arc generation between the cathode and the anode is increased. Therefore, argon thermal plasma has a lower average voltage with lower resistance than argon–nitrogen thermal plasma at the fixed current. Input power of 8.4 kW was sufficient to vaporize the Ga(NO_3_)_3_∙*x*H_2_O pellet due to its low vaporization temperature of 80 °C. Synthesized nanoparticles were attached on the inner surface of the reactor which was chilled by cooling water. The product was collected by scraping with a thin film for the post processing and analysis.

**Figure 2:**
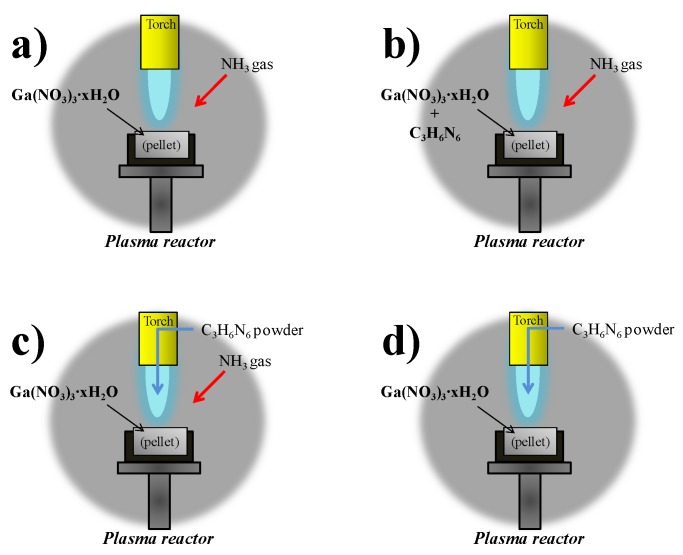
Detailed illustration of C_3_H_6_N_6_ injection methods in Plasma 1, 2, 3, 4, and 5; (**a**) Plasma 1; (**b**) Plasma 2; (**c**) Plasmas 3 and 4; (**d**) Plasma 5.

C_3_H_6_N_6_ powder was used in the three different procedures in Plasma 2, 3, 4, and 5. A detailed illustration of C_3_H_6_N_6_ injection methods is indicated in Figure 2a–d. C_3_H_6_N_6_ was mixed with Ga(NO_3_)_3_∙*x*H_2_O, they were pressed as the pellet precursor in Plasma 2. C_3_H_6_N_6_ powder was injected into the high temperature of the thermal plasma jet as powder through the anode electrode in Plasmas 3, 4, and 5. The molar ratio of Ga(NO_3_)_3_∙*x*H_2_O and C_3_H_6_N_6_ was controlled at 1:6 and 1:3 in Plasmas 3, 4, and 5. In these cases, NH_3_ gas was injected into the pellet as a nitridation source by the probe as in Figure 1. Ga(NO_3_)_3_∙*x*H_2_O was reacted with only C_3_H_6_N_6_ powder and without NH_3_ gas in Plasma 5, in order to check the possibility of nitridation by the nitrogen element of C_3_H_6_N_6_. The products from thermal plasma were annealed in a vacuum furnace (SH-TMFGF-50, Samheung Inc., Sejong, Korea) to eliminate contamination and to enhance the crystallinity. It was carried out at 850 °C for three hours.

**Figure 3:**
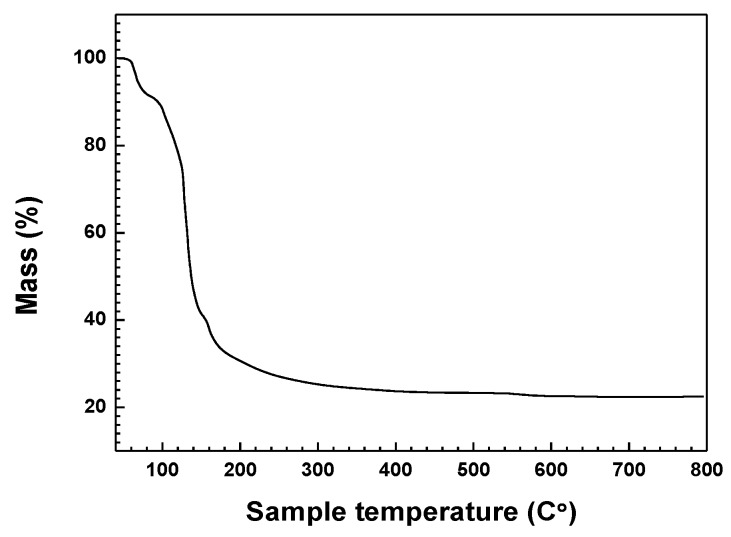
TGA (thermogravimetric analysis) of Ga(NO_3_)_3_∙*x*H_2_O raw material.

A TGA analysis result of Ga(NO_3_)_3_∙*x*H_2_O as raw materials according to temperature is indicated in Figure 3. Analysis was conducted from 40 °C to 800 °C with an increase in increments of 10 °C/min under a nitrogen atmosphere. An obvious mass reduction of approximately 67% occurred between 60 °C and 180 °C as is shown in Figure 3. Ga(NO_3_)_3_∙*x*H_2_O was converted to Ga_2_O_3_, H_2_O, and N_2_O_3_ gas with increasing temperatures, which is in accordance with previous studies [23]. Therefore, it can be assumed that the final product of Ga_2_O_3_ with a mass reduction of 78% would be produced at 800 °C. As a result, the estimated value of *x* in Ga(NO_3_)_3_∙*x*H_2_O was determined to be 32.1 based on the mass reduction from 12.45 mg at 40 °C to 2.80 mg at 800 °C. Accordingly, Ga(NO_3_)_3_∙*x*H_2_O is referred to as Ga(NO_3_)_3_∙32H_2_O in this work.

The crystallinity of the synthesized powder was observed by XRD (X-ray diffraction, DMAX 2500, Rigaku Co., Akishima, Japan) with Cu Kα source. Morphology and particle size were analyzed by FE-SEM (Field-emission scanning electron microscopy, S-4300, Hitachi Co., Tokyo, Japan) and FE-TEM (Field-emission transmission electron microscopy, JEM-2100F, Jeol, Japan). In addition, the elemental composition of particles was confirmed by an EDS (Energy dispersive spectroscopy) with SEM and TEM. The mean particles size was calculated from the variation of the hundreds of different synthesized particles in the FE-SEM images. TGA (thermogravimetric analysis, Diamond TG-DTA Lab system, Perkin Elmer, Waltham, MA, USA) was applied to investigate the atomic ratio of Ga(NO_3_)_3_∙*x*H_2_O as raw materials and the thermal behavior of particles synthesized by the thermal plasma and annealing procedure. Chemical bonding and nanostructure of particles synthesized by the thermal plasma and vacuum furnace method were analyzed by XPS (X-ray photoelectron spectroscopy, K-Alpha, Thermo scientific Inc., Waltham, MA, USA).

## 3. Results and Discussion

**Figure 4:**
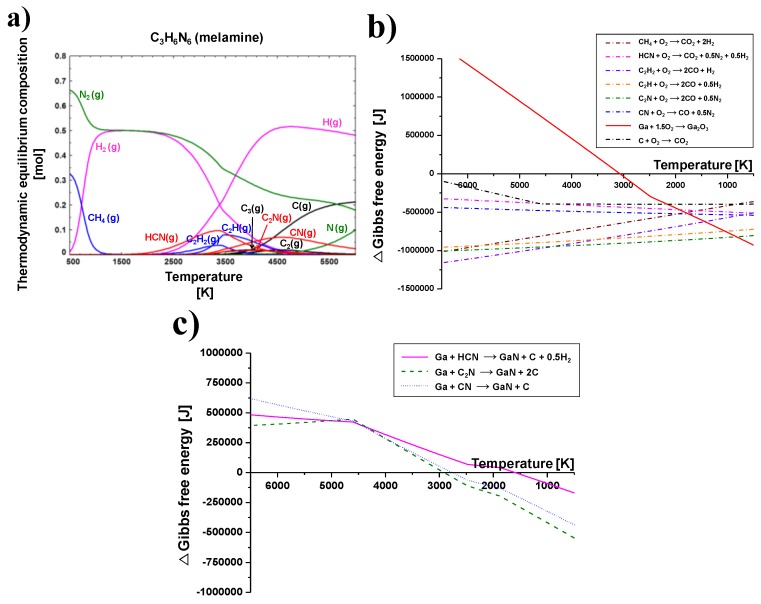
Thermodynamic equilibrium calculation at changes in temperature; (**a**) thermodynamic equilibrium composition of melamine; (**b**) change in Gibbs free energy during the oxygen capture reaction of C_3_H_6_N_6_ from Ga(NO_3_)_3_∙*x*H_2_O; and (**c**) change in Gibbs free energy of nitridation reaction by decomposed C_3_H_6_N_6_.

Figure 4a shows the thermodynamic equilibrium composition of melamine at changes in temperature from 500 to 6000 K. The calculation was conducted by a commercial software of FactSage (Ver. 6.4, CRCT&GTT, Canada and Germany). The purpose of thermodynamic equilibrium calculation was to expect a preferable chemical reaction and stable chemical species in a given temperature range. C_3_H_6_N_6_ containing carbon, hydrogen, and nitrogen elements can be decomposed and dissociated at about 350 °C. The melamine is converted to cyanides, hydrocarbons, and carbon in the high temperature plasma zone. These decomposed or dissociated C_3_H_6_N_6_ byproducts can react with the oxygen of a Ga(NO_3_)_3_∙32H_2_O precursor. Furthermore, the various exothermic gases such as CO, CO_2_, H_2_, N_2_, NO_2_, and the releasing of heat generated by the oxygen capture reaction with Ga(NO_3_)_3_∙32H_2_O and C_3_H_6_N_6_ promote the synthesis of GaN nanopowder.

The change in Gibbs free energy during oxygen capture reactions with byproducts converted by decomposition of the C_3_H_6_N_6_ which was injected into the thermal plasma jet are shown in Figure 4b. The graph consists of seven dotted lines and one solid line. The dotted lines indicate the oxidation by byproducts generated from C_3_H_6_N_6_ decomposition, while the solid line indicates the oxidation of gallium to Ga_2_O_3_. Oxidation reactions of hydrocarbons, cyanides, and carbon generated by the decomposition of C_3_H_6_N_6_ are thermodynamically spontaneous throughout the whole temperature range because Gibbs free energies are less than zero. Conversely, Ga_2_O_3_ can be produced spontaneously under 3100 K. However, oxidation of byproducts is still predominant at temperatures above 750 K owing to a greater amount of negative Gibbs free energy. Therefore, C_3_H_6_N_6_ powder is suitable to capture oxygen molecules of Ga(NO_3_)_3_∙32H_2_O before they oxidize with gallium, even though various byproducts could be produced from the decomposition of C_3_H_6_N_6_. For this reason, C_3_H_6_N_6_ powder was injected into the thermal plasma jet to facilitate the synthesis of GaN nanopowder. Moreover, the TGA analysis and thermodynamic equilibrium calculation revealed that six moles of C_3_H_6_N_6_ powder were needed, while only one mole of Ga(NO_3_)_3_∙32H_2_O was required to sufficiently convert oxygen from Ga(NO_3_)_3_∙32H_2_O into carbon oxides, nitrogen oxides, and hydrogen oxides.

Nitridation by the nitrogen element of C_3_H_6_N_6_ is considered in Figure 4c. The thermodynamic equilibrium is calculated for the nitridation reaction by HCN, C_2_N, and CN molecules. These components containing nitrogen elements were reacted with the Ga element at temperatures under 3000 K. Although ∆*G* of the three nitridation reactions have negative values, they are lower than those of the oxidation reaction in Figure 4b. ∆*G* values for the oxidation reaction of HCN, C_2_N, and CN at about 3000 K are under 500,000 J, as shown in Figure 4b. Those values for their nitridation reaction at approximately 3000 K are nearly zero. In other words, oxidation reaction is absolutely superior compared to the nitridation reaction at all temperature ranges. Therefore, the byproducts, which are converted from the decomposition of C_3_H_6_N_6_, usually react with the oxygen elements of the Ga(NO_3_)_3_∙32H_2_O raw material.

**Figure 5:**
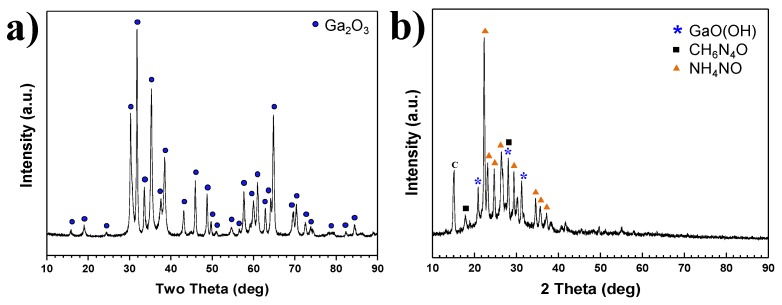
XRD (X-ray diffraction) pattern of product synthesized by thermal plasma; (**a**) Plasma 1 by Ga(NO_3_)_3_∙32H_2_O and NH_3_ without C_3_H_6_N_6_; (**b**) Plasma 2 by Ga(NO_3_)_3_∙32H_2_O and C_3_H_6_N_6_ pellet with NH_3_ gas.

XRD patterns of products synthesized in Plasma 1 and 2 conditions are shown in Figure 5a,b, respectively. For Plasma 1, only a Ga(NO_3_)_3_∙32H_2_O pellet was used with 10 L/min NH_3_ to synthesize GaN nanopowder without C_3_H_6_N_6_. The plasma jet was generated by argon and nitrogen mixed gas in a conventional synthesis process of nitride materials by thermal plasma. All peaks shown in Figure 5a correspond to Ga_2_O_3_. The results revealed that Ga(NO_3_)_3_∙32H_2_O is not nitrided solely by NH_3_ gas, which is a typical nitrogen source for the nitridation reaction. Ionized nitrogen gas which is produced by the generating thermal plasma jet was not suitable for the nitridation of Ga(NO_3_)_3_∙32H_2_O. Ga(NO_3_)_3_∙32H_2_O has a low evaporation temperature and is rapidly vaporized in the high temperature environment of the thermal plasma jet. However, nitridation of Ga did not occur, and oxidation of Ga was completed by the presence of abundant oxygen elements from the precursor itself. NH_3_ gas usually decomposes into N_2_ and H_2_ gases at high temperatures. As shown in the following chemical reactions, the oxidation of Ga dominates over the reaction with N_2_ and NH_3_ to GaN.

Ga + 0.5 N_2_ → GaN     ∆H_298K_: −109.6 kJ (1)

Ga + NH_3_ → GaN + 1.5 H_2_     ∆H_298K_: −63.7 kJ (2)

2 Ga + 1.5 O_2_ → Ga_2_O_3_     ∆H_298K_: −1089.1 kJ (3)

These findings indicate that the nitridation of Ga(NO_3_)_3_∙32H_2_O is difficult without a substance that can consume oxygen as a reductant. Therefore, C_3_H_6_N_6_ powder was used as a reductant to oxidize with the abundant oxygen elements of the Ga(NO_3_)_3_∙32H_2_O precursor.

C_3_H_6_N_6_ powder was used as a pellet by compressing it with Ga(NO_3_)_3_∙32H_2_O as a precursor in Plasma 2. The pellet consisting of Ga(NO_3_)_3_∙32H_2_O and C_3_H_6_N_6_ was vaporized by a thermal plasma jet. NH_3_ gas was injected into the pellet at 3 L/min. The XRD pattern of synthesized product at Plasma 2 conditions is indicated in Figure 5b. The peaks are analyzed as gallium hydroxide (GaO(OH)), carbohydrazide (CH_6_N_4_O), and ammonium nitrate (NH_4_NO). The vaporized Ga elements were oxidized, and C_3_H_6_N_6_ was not applied in sufficient amounts to act as a reductant. Unlike the experiment involving Ga(NO_3_)_3_∙32H_2_O and NH_3_ gas without C_3_H_6_N_6_, GaO(OH) was produced. Products including C-H-O bonding or ammonium ions were produced in the experiment using a pellet mixed with C_3_H_6_N_6_ and Ga(NO_3_)_3_∙32H_2_O. It was assumed that C_3_H_6_N_6_ could not be used to capture oxygen when it was mixed with Ga(NO_3_)_3_∙32H_2_O in a pellet. Although C_3_H_6_N_6_ affects the product, the effect is not sufficient to nitrate the gallium. Ga(NO_3_)_3_∙32H_2_O was vaporized above 80 °C. The vaporizing temperature of C_3_H_6_N_6_ is 345 °C. The difference of vaporization temperatures for the two raw materials should be considered to prevent oxidation of the Ga element. The useful reductants generated by vaporization of C_3_H_6_N_6_ have to react with the gallium element before oxidation. In order to vaporize C_3_H_6_N_6_ early, it was injected into the higher temperature region of the thermal plasma.

**Figure 6:**
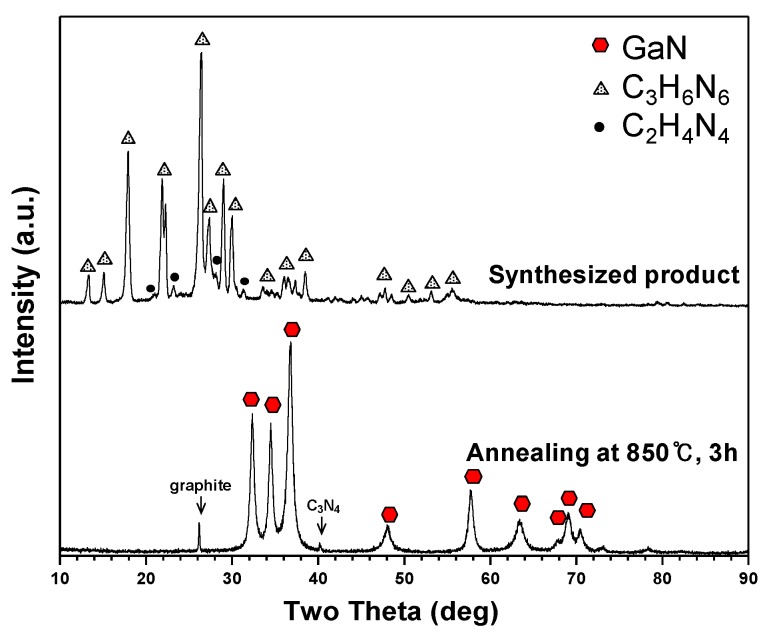
XRD patterns of products synthesized from a Ga(NO_3_)_3_∙32H_2_O pellet and C_3_H_6_N_6_ powder injection with NH_3_ gas by the thermal plasma process in Plasma 3.

C_3_H_6_N_6_ powder was fed solely into a high temperature thermal plasma jet through two nozzles inside the anode electrode to enable more active decomposition. Ga(NO_3_)_3_∙32H_2_O has an evaporation temperature below 100 °C, and rapidly vaporized when it contacted the high temperature thermal plasma jet. The molar ratio of Ga(NO_3_)_3_∙32H_2_O and C_3_H_6_N_6_ was controlled at 1:6 and 1:3 in Plasma 3 and 4 conditions. Initially, C_3_H_6_N_6_ powder was injected at a 1:6 molar ratio according to the calculation from the TGA analysis of Ga(NO_3_)_3_∙32H_2_O raw materials. This molar ratio was sufficient to oxidize C_3_H_6_N_6_ from the oxygen element of the Ga(NO_3_)_3_∙32H_2_O. The graph in Figure 6 shows XRD patterns of the product synthesized in Plasma 3. The main peaks correspond to C_3_H_6_N_6_ and other peaks reflect C*_x_*H*_y_*N*_z_* bound byproducts such as melam, melem and melon. C_3_H_6_N_6_ can be converted into other derivatives by thermal condensation [24,25,26,27]. The synthesized product was subjected to annealing in a vacuum furnace to enhance the GaN crystallinity and eliminate the residual C_3_H_6_N_6_. The XRD pattern of annealed nanopowder is indicated in Figure 6. Distinct peaks of GaN were observed after annealing at 850 °C for three hours. Weak graphite and carbon nitride (C_3_N_4_) peaks were also observed. It was estimated that the synthesized GaN nanopowder was not accurately detected in the XRD pattern due to its low crystallinity and low quantity.

**Figure 7:**
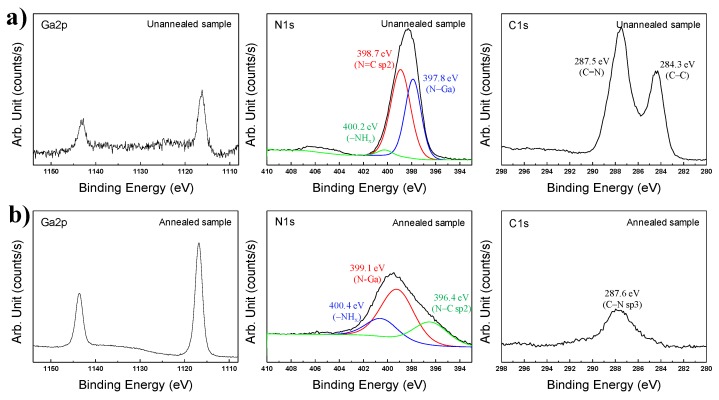
XPS (X-ray photoelectron spectroscopy analysis) of nanopowder synthesized from a Ga(NO_3_)_3_∙32H_2_O pellet and C_3_H_6_N_6_ powder injection with NH_3_ gas by the thermal plasma process in Plasma 3; (**a**) before and (**b**) after annealing.

This result was reliable as confirmed by the Ga–N chemical bonding by XPS results shown in Figure 7. The Ga2p, N1s, and C1s orbital graphs are indicated in Figure 7a,b. In the Ga2p graphs of Figure 7a, Ga–N bonding peaks of Ga2p_(1/2)_ and Ga2p_(3/2)_ were identically observed at 1143.0 eV and 1116.2 eV [26,28,29,30,31]. Although peak intensities of Ga2p_(1/2)_ and Ga2p_(3/2)_ in Figure 7a are weak, the two peaks could be accurately observed in the Ga2p graph. This result demonstrated that GaN was definitely synthesized by the thermal plasma, but was not detected in the XRD pattern due to its low crystallinity. The N=C sp2 binding peak is high (398.7 eV) in the N1s graphs of Figure 7. This peak was caused by residual C_3_H_6_N_6_ and its derivatives after synthesis using thermal plasma [32]. An N–Ga bonding peak was observed at 397.8 eV. Amino functional groups having (–NH*_x_*) were indicated at 400.2 eV by residual C_3_H_6_N_6_ and it derivatives. In the C1s graph, C=N and C–C binding peaks were present at 287.5 and 284.3 eV, respectively, which was attributed to the remaining C_3_H_6_N_6_. Additionally, the intensity of the C=N bonding peak was higher than that of the C-C bonding peak [32].

Figure 7b shows XPS analysis results of annealed nanopowder after thermal plasma synthesis in Plasma 3. XRD and TEM analysis revealed that the GaN synthesized by the thermal plasma had enhanced crystallinity after annealing. After annealing at 850 °C under rough vacuum pressure for three hours, the peak intensities of Ga–N bonding were increased to levels which were significantly higher than before the annealing step. Ga–N bonding peaks of Ga2p_(1/2)_ and Ga2p_(3/2)_ were observed at 1143.5 eV and 1116.7 eV, respectively [26,28,29,30,31]. However, the N1s graphs all show different peak trends, as is shown in Figure 7a. Following annealing of the product synthesized in Plasma 3, the peaks appeared at 400.4 and 396.4 eV in N1s, which can be observed in Figure 7b. This peak was attributed to nitrogen in the –NH*_x_* and N–C sp2 bond from C_3_N_4_ generated from the conversion of residual C_3_H_6_N_6_ [33,34]. A deconvoluted N–Ga bonding peak was observed at 399.1 eV [27]. This peak was shifted compared to pre-annealing due to the sufficient levels of nitrogen of synthesized GaN by decomposition of residual C_3_H_6_N_6_. In the C1s graph of Figure 7b, a C–N sp3 bonding peak was produced at 287.6 eV. C–C bonding peaks are deconvoluted at 289.8 and 284.8 eV [33,34].

**Figure 8:**
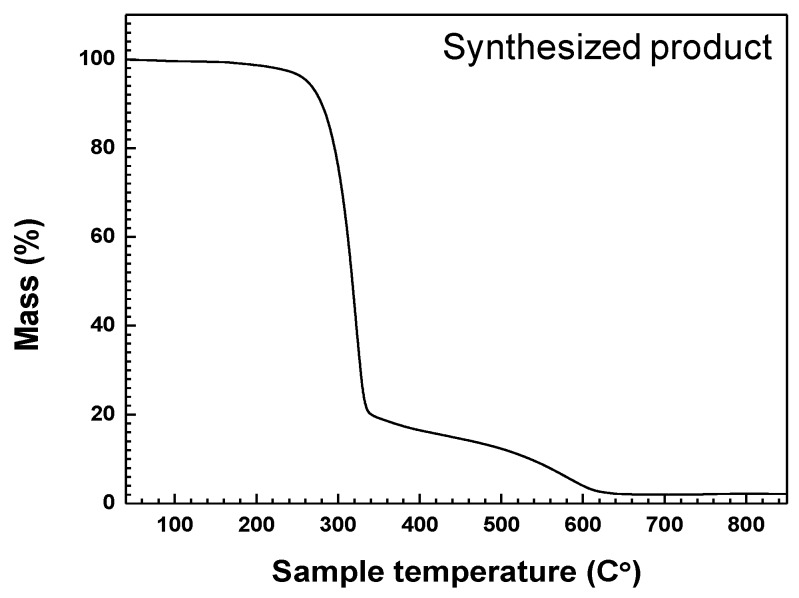
TGA analysis of product synthesized from a Ga(NO_3_)_3_∙32H_2_O pellet and C_3_H_6_N_6_ powder injection with NH_3_ gas by the thermal plasma process in Plasma 3.

The results of TGA analysis of product synthesized under the Plasma 3 operating conditions are indicated in Figure 8. The temperature of the crucible containing the synthesized product powder was increased from 40 °C to 850 °C at 10 °C/min intervals. The atmosphere was filled with a nitrogen gas. A total mass reduction of up to 98% of weight occurred. The “as prepared product” after TGA analysis was considered to consist of GaN and a small amount of C_3_N_4_. As reported in previous studies [26,33,34], C_3_N_4_ can be synthesized at high temperatures (>600 °C) by slowly heating C_3_H_6_N_6_ alone. The mass reduction curve had a high gradient from about 300 °C to 650 °C. The residual C_3_H_6_N_6_ and derivatives undergo thermal degradation and decomposition at temperatures in excess of 300 °C. Based on the mass maintenance at above 620 °C, the temperature for annealing to enhance the crystallinity of synthesized GaN nanopowder was set at 850 °C. Since the melting point of GaN is above 2500 °C, synthesized GaN nanopowder was not melted or vaporized by the annealing.

**Figure 9:**
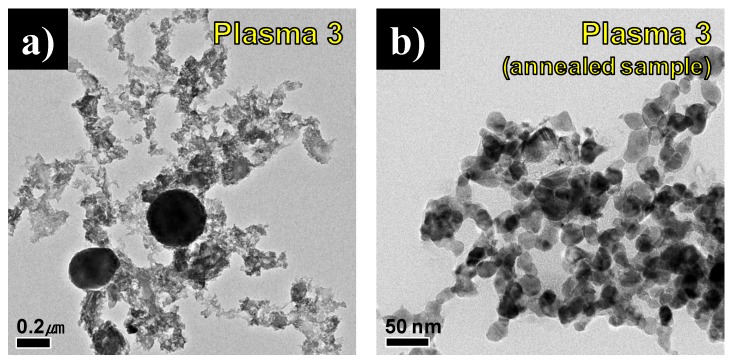

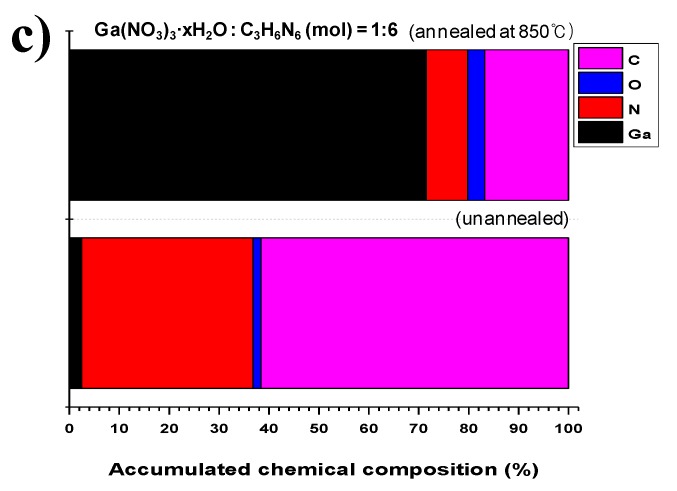
FE-TEM (field-emission scanning electron microscopy) and EDS (energy dispersive spectroscopy) results of product synthesized from a Ga(NO_3_)_3_∙32H_2_O pellet and C_3_H_6_N_6_ powder injection with NH_3_ gas by the thermal plasma process in Plasma 3; FE-TEM images of (**a**) before and (**b**) after annealing; (**c**) TEM-EDS results.

Figure 9a,b shows FE-TEM images of the synthesized product before and after annealing in Plasma 3 conditions. Small particles under 100 nm and spherical large particles of about 200 nm were mixed in Figure 9a. After annealing, the large spherical particles were no longer present, and only nano-sized round particles which had an average particle size of 21.63 nm remained, as is shown in Figure 9b. It is believed that the large spherical particles are the residual C_3_H_6_N_6_ precursor. Figure 9c shows TEM-EDS results before and after annealing in Plasma 3. The Ga element was detected as about 2.5% of weight before annealing. It was revealed that melamine powder remained in micro-sized particles. The content of Ga elements was increased dramatically from 2.45% to 71.6% of weight after annealing. This analysis demonstrates that the purification was completed and residual melamine powder was eliminated by the heat treatment of the vacuum furnace. As shown by the XRD patterns in Figure 6b, a small amount of carbon and carbon nitride was generated after annealing because the treatment was done under vacuum conditions. The vaporized melamine converted as a byproduct with gallium nitride nanoparticles.

**Figure 10:**
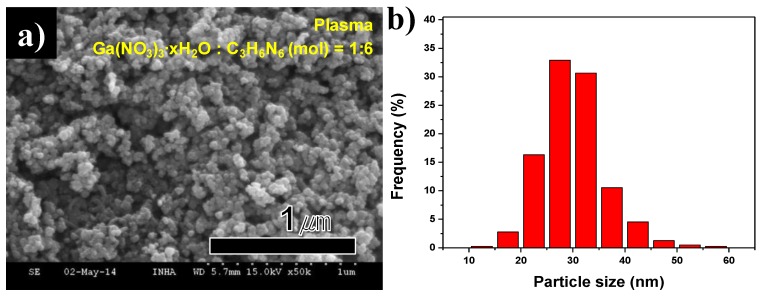
(**a**) FE-SEM image and (**b**) size distribution of synthesized GaN nanopowder after annealing in Plasma 3.

FE-SEM images and size distribution of synthesized GaN nanopowder are indicated in Figure 10a,b. The large residual C_3_H_6_N_6_ particles were removed, but the uniform GaN nanoparticles remained, as is shown in Figure 10a. The particle size distribution was arranged by measurement of each particle in the FE-SEM frame, as is seen in Figure 10b. The size distribution of synthesized nanoparticles was analyzed by FE-SEM images. Hundreds of nanoparticles were measured with particles sizes varying from 10 to 60 nm. The mean particle size was 29.8 nm. The uniform-sized particle GaN nanopowder was synthesized by thermal plasma and an additional annealing process.

**Figure 11:**
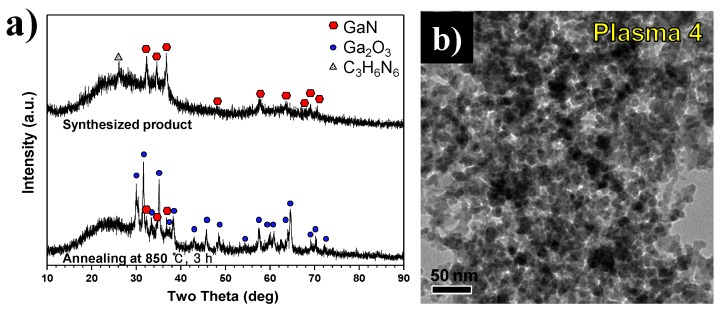
(**a**) XRD patterns before and after annealing and (**b**) FE-TEM image of unannealed product from a Ga(NO_3_)_3_∙32H_2_O pellet and C_3_H_6_N_6_ powder injection with NH_3_ gas by the thermal plasma process in Plasma 4.

To reduce residual C_3_H_6_N_6_ present in the product generated by the thermal plasma, the ratio of Ga(NO_3_)_3_∙32H_2_O and C_3_H_6_N_6_ was decreased to 1:3 under the operating conditions used to generate Plasma 4. XRD patterns of product synthesized under these conditions are shown in Figure 11a. The upper pattern is for synthesized nanopowder by thermal plasma while the bottom pattern is for annealed nanopowder at 850 °C for three hours. In the upper XRD pattern, the main peak of C_3_H_6_N_6_ was observed at a weak 26°, while main peaks of GaN were found at 32°, 34°, and 38°. However, Ga_2_O_3_ peaks appeared after annealing in the vacuum furnace at 850 °C for three hours. These findings demonstrate that the oxygen capture reaction of Ga(NO_3_)_3_∙32H_2_O by C_3_H_6_N_6_ could have negative effects as the amount of C_3_H_6_N_6_ decreases.

Figure 11b shows FE-TEM images of synthesized nanopowder by thermal plasma, when the molar ratio of Ga(NO_3_)_3_∙32H_2_O and C_3_H_6_N_6_ of 1:3 is adopted in Plasma 4 conditions. The product synthesized in Plasma 4 primarily consisted of fine particles which had an average particle size of 9.25 nm. In the EDS result in Table 2, the content of the Ga element is higher, about 63% of weight, than that of the synthesized nanopowder in Plasma 3 without additional annealing. However, the oxygen content is high at 23% of weight due to incomplete nitridation and generated gallium oxide. The degree of Ga oxidation increased as the molar ratio of injected C_3_H_6_N_6_ decreased. Therefore, injection of excessive C_3_H_6_N_6_ is effective for preventing the oxidization of Ga. Moreover, C_3_H_6_N_6_ aids in the complete synthesizing of GaN nanopowder. However, annealing is required to eliminate the remaining C_3_H_6_N_6_.

**Figure 12:**
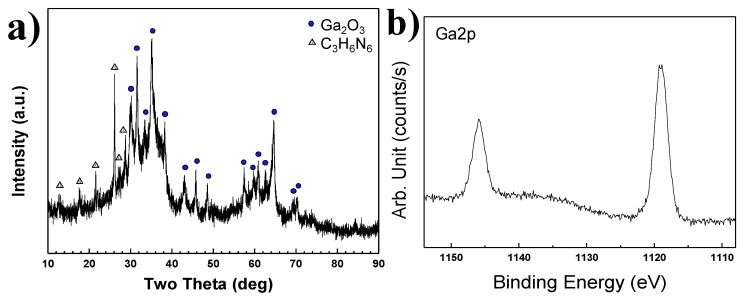
(**a**) XRD patterns and (**b**) XPS analysis of products synthesized from a Ga(NO_3_)_3_∙32H_2_O pellet and C_3_H_6_N_6_ powder injection without NH_3_ gas by the thermal plasma process in Plasma 5.

C_3_H_6_N_6_ was used as a reductant to prevent oxidation of gallium. However, it also consists of an abundant source of nitrogen. Therefore, a nitridation reaction using only C_3_H_6_N_6_ powder, without the addition of NH_3_ is needed to confirm the complete synthesis of GaN nanopowder. In previous experiments, NH_3_ gas was injected into the Ga(NO_3_)_3_∙32H_2_O pellet as a nitridation source. A Ga(NO_3_)_3_∙32H_2_O pellet was reacted with only C_3_H_6_N_6_ powder without NH_3_ gas in Plasma 5 conditions. C_3_H_6_N_6_ powder was injected through two nozzles of an anode electrode as in Plasma 3. The molar ratio of Ga(NO_3_)_3_∙32H_2_O and C_3_H_6_N_6_ was set at 1:6. The XRD pattern of synthesized nanopowder by reacting Ga(NO_3_)_3_∙32H_2_O and C_3_H_6_N_6_ without NH_3_ gas is indicated in Figure 12a. The intensities of all peaks were weak. The observed peaks corresponded with gallium oxide and melamine. When the NH_3_ gas was injected into the reactor at the same molar ratio of Ga(NO_3_)_3_∙32H_2_O and C_3_H_6_N_6_ in Plasma 3, only residual C_3_H_6_N_6_ peaks were detected in the XRD pattern in Figure 6. Low crystallinity GaN was synthesized. However, Ga_2_O_3_ peaks were detected with residual C_3_H_6_N_6_ in Plasma 5 of Figure 12a. It was revealed that the nitridation reaction did not occur with the nitrogen element of C_3_H_6_N_6_ powder. Figure 12b shows XPS analysis results of synthesized nanopowder in Plasma 5. Ga-N bonding peaks as Ga2p_(1/2)_ and Ga2p_(3/2)_ were identically observed at 1143.0 eV and 1116.2 eV. However, two peaks were observed at 1145.9 eV and 1119.3 eV. Analysis of these peaks confirmed Ga–O chemical bonding. An increase of binding energy from Ga2p electrons was observed due to Ga–O chemical bonding. The nitrogen element has a less electronegative property than the oxygen element. Therefore, the binding energy of Ga–O chemical bonding is higher than that of Ga–N chemical bonding [30].

C_3_H_6_N_6_ was decomposed and converted to cyanides, hydrocarbons, and carbon in the high temperature of the thermal plasma jet. Among these, only cyanides have nitrogen elements. However, the nitridation reaction by cyanide molecules is an inferior chemical reaction compared with oxidation. It was examined using the thermodynamic equilibrium calculation in Figure 4c.

Overall, these findings indicate that GaN nanopowder was synthesized from Ga(NO_3_)_3_∙32H_2_O and C_3_H_6_N_6_ by thermal plasma, despite low crystallinity. Its crystallinity can be enhanced by annealing in a vacuum furnace. Moreover, if the annealing can be completed at atmospheric pressure in an inert atmosphere, not by rough vacuum, production of carbon dioxide or carbon nitride can be controlled.

## 4. Conclusions

GaN nanopowder was synthesized by a thermal plasma process. Ga(NO_3_)_3_∙*x*H_2_O was used as the raw material. At first, it was discovered that Ga(NO_3_)_3_∙*x*H_2_O was not nitrided by a solely conventional NH_3_ nitridation source and that it is converted into Ga_2_O_3_. It required a reductant to prevent oxidation to Ga_2_O_3_ instead of GaN. Therefore, C_3_H_6_N_6_ powder was injected into the high temperature region of the thermal plasma jet through an anode electrode. The molar ratio of injected Ga(NO_3_)_3_∙*x*H_2_O and C_3_H_6_N_6_ was controlled at 1:6 and 1:3. GaN nanopowder with low crystallinity and residual C_3_H_6_N_6_ was synthesized by the thermal plasma process. Crystallinity of the synthesized GaN nanopowder was further enhanced after annealing at 850 °C for three hours in a vacuum furnace. The size of synthesized GaN nanopowder is distributed from 10 to 60 nm, and mean particle size is calculated to be 29.8 nm. In order to confirm the nitridation of Ga(NO_3_)_3_∙*x*H_2_O by C_3_H_6_N_6_ reductant, the Ga(NO_3_)_3_∙*x*H_2_O was reacted with C_3_H_6_N_6_ without NH_3_ gas, and Ga(NO_3_)_3_∙*x*H_2_O precursor was oxidized to Ga_2_O_3_. Therefore, GaN nanopowder was successfully synthesized from Ga(NO_3_)_3_∙32H_2_O and C_3_H_6_N_6_ powders with NH_3_ gas by a thermal plasma process. Furthermore, the additional annealing step was required to enhance its crystallinity. It was speculated that the production of carbon dioxide or carbon nitride during the annealing step could be controlled as anneal in an inert atmosphere. The synthesis of GaN crystalline nanopowder is difficult to achieve using conventional production methods. This research demonstrates the potential to synthesize GaN nanopowder through a thermal plasma process, from raw materials comprising abundant oxygen elements.

## Figures and Tables

**Table 1:** Operating conditions for the synthesis of GaN powder by thermal plasma.

Experiment No.		Plasma 1	Plasma 2	Plasma 3	Plasma 4	Plasma 5
**Condition of precursors**	**Weight of Ga(NO_3_)_3_∙*x*H_2_O**	6 g				
	**Weight of C_3_H_6_N_6_**	-	15 g (pellet)	15 g (powder injection)	8.8 g	15 g (powder injection)
	**Molar ratio of Ga(NO_3_)_3_∙*x*H_2_O and C_3_H_6_N_6_**	-	1:6	1:6	1:3	1:6
**Condition of plasma generating**	**Flow rate of plasma forming gas**	13 L/min Ar + 2 L/min N_2_	13 L/min Ar			
	**Flow rate of reactive NH_3_ gas**	10 L/min	3 L/min	3 L/min	3 L/min	-
	**Flow rate of carrier gas**	-	-	3 L/min N_2_	3 L/min N_2_	3 L/min N_2_
	**Plasma input power**	12.6 kW (300 A, 42 V)	8.4 kW (300 A, 28 V)			

**Table 2:** Elemental composition of product synthesized from a Ga(NO_3_)_3_∙32H_2_O pellet and C_3_H_6_N_6_ powder injection with NH_3_ gas by the thermal plasma process in Plasma 4 (all results in % of weight).

Spectrum	C	N	O	Ga	Total
Spectrum 1	8.70	3.51	23.04	64.76	100.00
Spectrum 2	12.60	4.78	23.34	59.28	100.00
Spectrum 3	9.05	1.73	23.40	65.82	100.00
Mean	10.11	3.34	23.26	63.28	100.00
